# Case report: A rare case of massive peripheral nerve melanotic schwannoma and review of the literature

**DOI:** 10.3389/fneur.2023.1103604

**Published:** 2023-01-26

**Authors:** Han Wang, Lin Shi, Tong Tian, Hang Xian, Zhao Song, Rui Cong, Rui Zhao

**Affiliations:** ^1^Department of Orthopedics, Air Force Medical Center, Beijing, China; ^2^Department of Orthopedics, Xijing Hospital, Air Force Military Medical University, Xi'an, China

**Keywords:** melanotic schwannoma, peripheral nerves, brachial plexus, pathological diagnosis, neuropathic pain

## Abstract

Melanotic schwannoma is a rare tumor with indeterminate biologic behavior and varying treatment recommendations. Just about 200 cases have been reported worldwide, in which occurred in peripheral nerves has even less reported. Due to the lack of cognition of melanotic schwannoma, it is easy to be misdiagnosed and mistreatment in primary hospitals. Herein, we presented a case of massive melanotic schwannoma growing in the brachial plexus of an elderly male patient. First, the patient underwent a left forearm tumor resection in the local primary hospital because a painless lump was found there in 2017, of which details remain unclear. After this operation, the patient developed the symptoms of left median nerve injury. Thus, he came to our hospital and underwent a second operation. During this operation, we found that a part of the median nerve was absent at the left forearm, and the remanent median nerve, from the broken end to the elbow, was totally turned black, which was accompanied by petroleum-like exudate. Losing the opportunity for nerve repair, the black nerve was removed extensively and thoroughly. Postoperative pathological diagnosis revealed that the tumor was melanotic schwannoma. Then 4 years later, the tumor recurrence again, which led to the paralysis of the whole left arm and severe nerve pain, and the pulmonary metastasis of the tumor was detected at the same time. The black nerve was resected again in our hospital, and the nerve pain was partially relieved after the operation. To the best of our knowledge, it is the first time to report a melanotic schwannoma case that happened in the peripheral nerve trunk and then spread to the whole brachial plexus. There were many questions that worthy of discussion could be invited from this case, and we analyzed and discussed them based on the relevant literature. In conclusion, we reported a rare case of melanotic schwannoma that happened in the brachial plexus and illustrated the problems of the diagnosis and treatment of it based on the analysis of the relevant literature, which is helpful for the cognition of this rare nerve tumor.

## Introduction

Melanotic schwannoma (MS) is a rare tumor of the nervous system, which accounts for < 1% of nervous system tumors. However, its specific incidence has not been reported yet. Since it was first reported by Hodson and J. J. in 1961, only about 200 cases have been reported worldwide (1). With the improvement in detection methods, the reports on MS have gradually increased in recent years. It has been reported that MS mainly occurs in the skin, organs, and spinal cord, but rarely in the peripheral nerve trunk. Many studies have pointed out that MS can be divided into the psammomatous type and the non-psammomatous type. Psammomatous melanotic schwannoma (PMS) is considered to have a potential malignant tendency, which can cause distant metastasis (2). Due to the lack of awareness of this disease, it is prone to misdiagnosis and mistreatment during diagnosis and treatment, especially in grass-roots hospitals. Therefore, we reported a rare case of recurrent giant MS in the peripheral nerve and discussed the diagnosis and treatment methods of this rare disease based on the relevant literature.

## Case report

A 62-year-old elderly Chinese man visited our hospital because of left hand numbness for 1 year after resection of a tumor in the forearm, accompanied by a tumor of the left hand for half a year. In 2016, the patient found a painless tumor at the left distal forearm of no obvious cause, without left hand numbness, movement disorders, or muscular atrophy. The tumor of the left forearm was resected in a local grass-roots hospital, with an unknown specific surgical condition and no definite postoperative diagnosis. After the surgery, the patient developed numbness of three and a half fingers on the radial side of the left hand, accompanied by the inflexibility of the left thumb. About half a year later, the patient found another painless tumor in the hypothenar region of the left hand, accompanied by progressive tumor enlargement, weakness of the left thumb, and progressive aggravation of dyskinesia. The patient visited our hospital for further treatment. Considering the recurrent tumor adjacent to the site of tumor resection, accompanied by iatrogenic injury of the median nerve, the patient was admitted to our department with “a tumor of the left palm, and left median nerve injury”. After admission, the preoperative examination was completed, and magnetic resonance imaging (MRI) of the left hand suggested a space-occupying lesion in the hypothenar region of the left hand, with a size of about 5 × 3 × 2 cm, and low T1 and high T2 signals.

**Figure 1:**
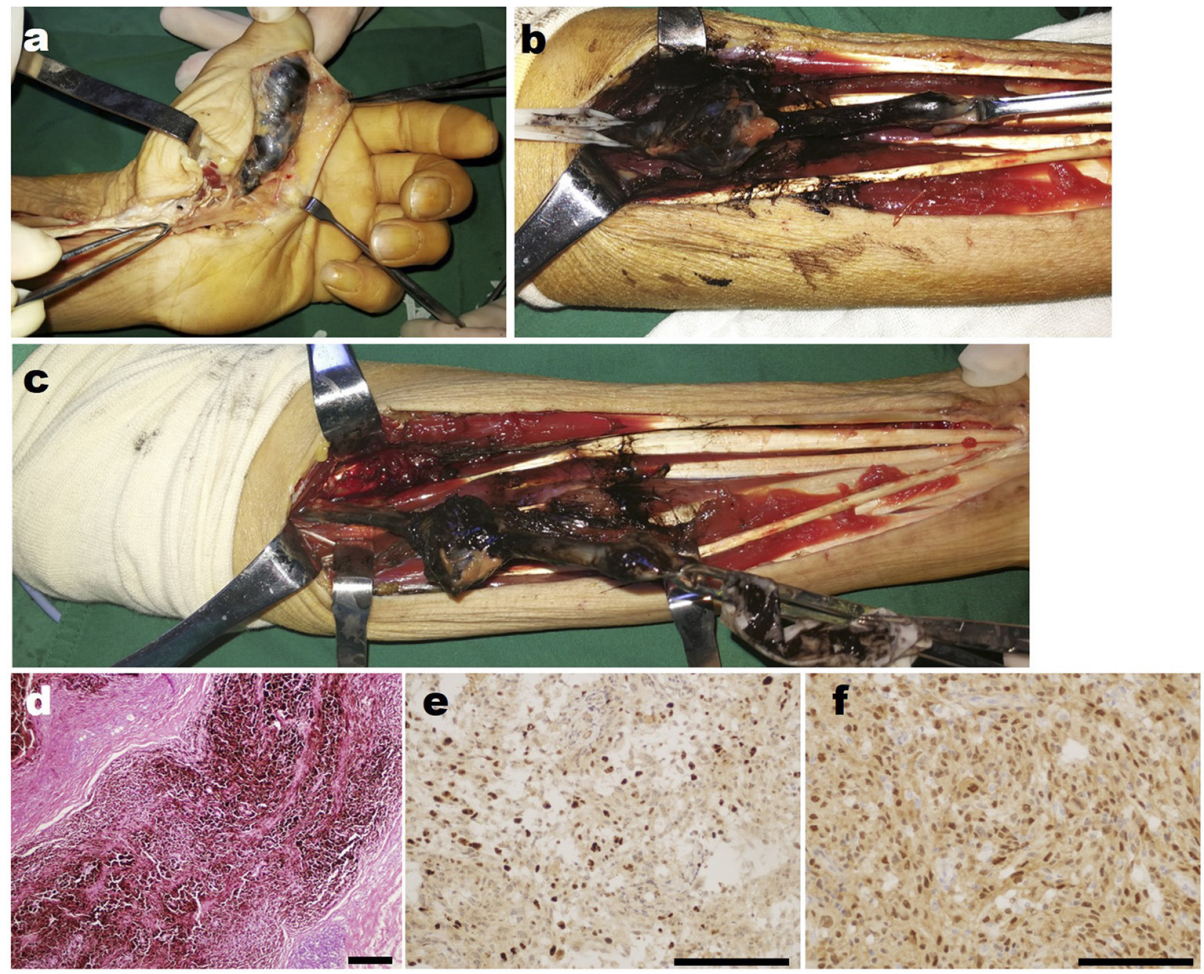
Intra-operative findings of the patient's first operation in our hospital. **(a)** The intraoperative photograph of the mass in the left hand of the patient. **(b)** The close-up detailed photograph of the mass in the left forearm shows the petroleum-like exudate. **(c)** The overall photographs of the mass in the left forearm of the patient. **(d)** The representative HE staining image of the resected tumor. **(e)** The representative Ki67 immunohistochemical staining image of the resected tumor. **(f)** The representative S100 immunohistochemical staining image of the resected tumor. Scar bar, 100 μm.

After preoperative preparation, tumor resection of the left hand and biopsy and median nerve exploration and repair in the left forearm were performed. However, the intraoperative findings were unexpected. A spindle-shaped enlarged mass was found in the thenar region of the left hand, which was completely black and connected with the digital nerve (Figure 1a). When the mass was cut open, melanotic nerve fibers and petroleum-like exudate were observed. A similar phenomenon also occurred in the left forearm. Intraoperatively, findings showed a defect of the left median nerve of about 10 cm in the distal forearm, and melanosis and thickening at about 13 cm from the proximal end of the nerve defect, in the center of which there was a quasi-circular mass, with a diameter of about 4 cm. After cutting open the mass, melanotic nerve fibers and black tumor-like hyperplasia were found, accompanied by black petroleum-like exudate (Figures 1b, c). According to the morphological features, MS was diagnosed intraoperatively, and the diseased nerves that were explored were resected completely and extensively. The tumor was extendedly resected at 2 cm from the tumor boundary trying to ensure no melanotic tumor cells at the incisal margin. Because of the lack of nerve repair condition, median nerve repair was abandoned in this surgery. After explaining and communicating with the patient about the condition, the patient and his family members expressed understanding and returned home for resting after the wound healed. Postoperative pathological confirmed MS and it was pathologically manifested as massive melanin deposition in tumor cells, and immunohistochemical staining suggested S100 and Ki67-positive, indicating that this tumor has a potential malignant tendency (Figures 1d–f).

**Figure 2:**
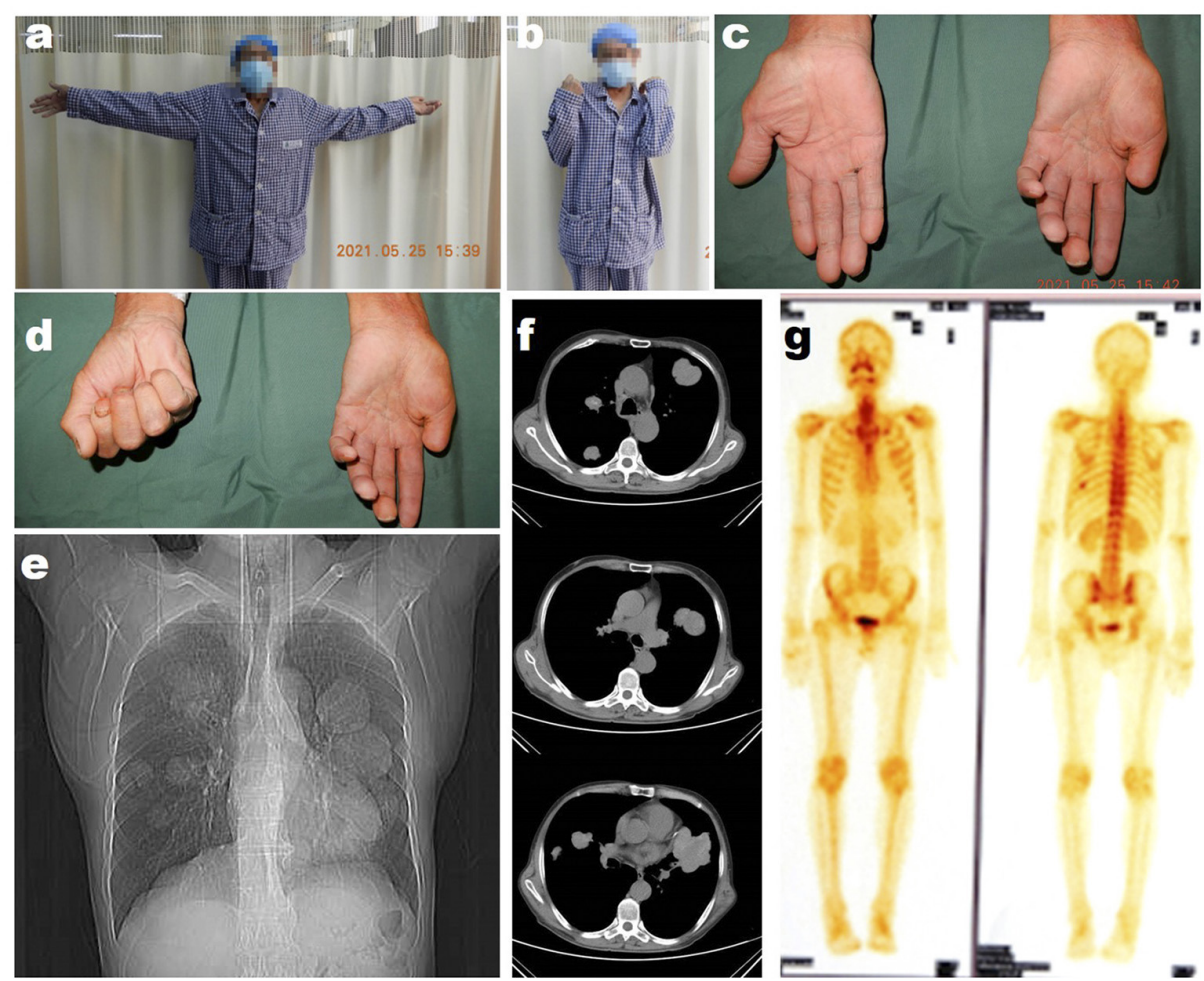
The preoperative examination of the patient's second operation in our hospital. **(a)** The photograph of the patient trying to abduct his bilateral shoulders. **(b)** The photograph of the patient trying to flex his bilateral elbows. **(c)** The photograph of the patient trying to extend his bilateral fingers. **(d)** The photograph of the patient trying to flex his bilateral fingers. **(e)** The radiological image of the patient's chest. **(f)** The representative CT images of the patient's chest. **(g)** The bone scan images of the patient.

**Figure 3:**
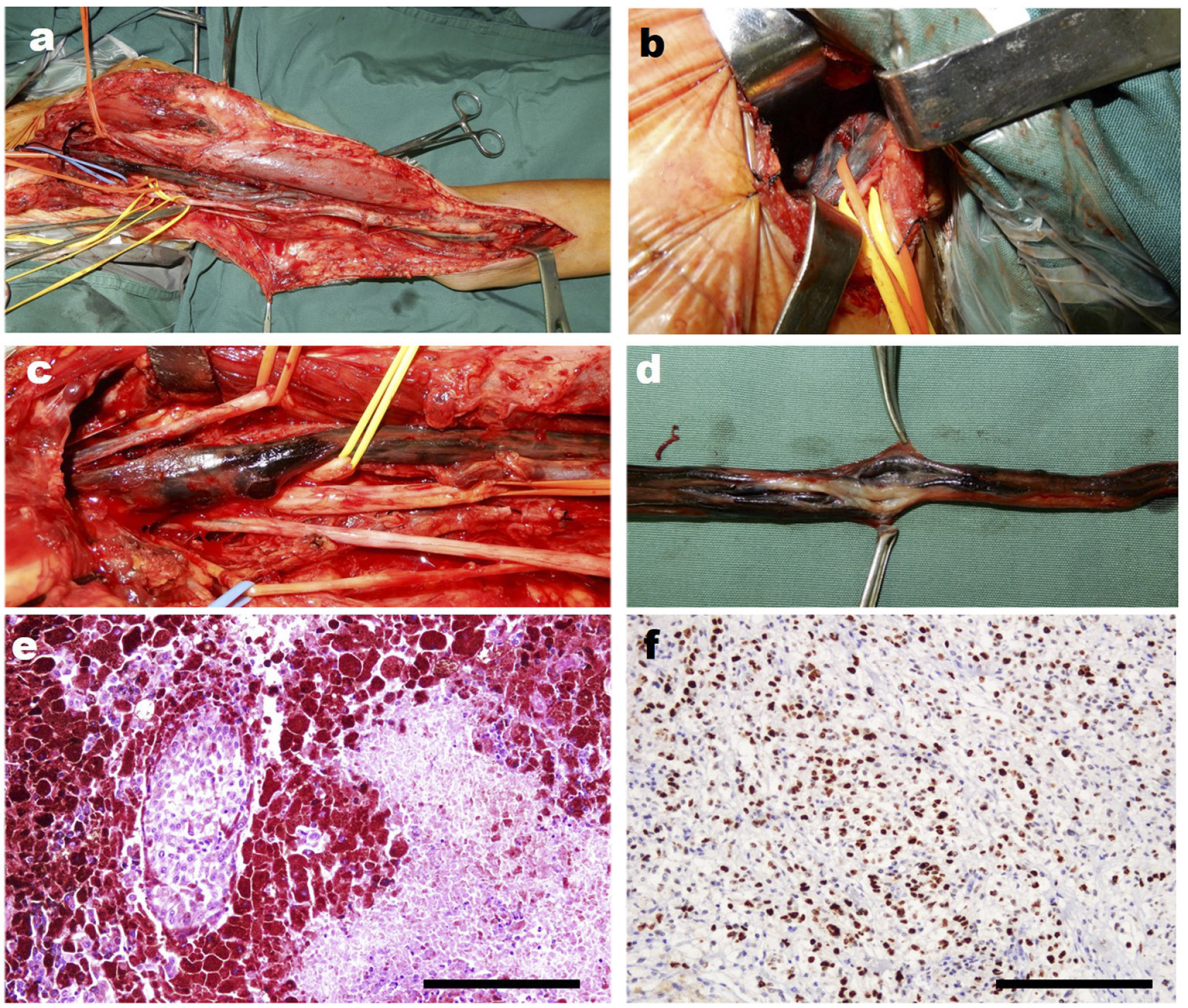
The intra-operative photographs of the patient's second operation in our hospital. **(a)** The overall photograph of the mass in the patient's left arm. **(b)** The intraoperative photograph of the mass in trunks of the brachial plexus. **(c)** The close-up detail photograph of the black changed the nerves of the patient. **(d)** The close-up detail photograph of the black changed nerve shows the change of the never fibers intra epineurium. **(e)** The representative HE staining image of the resected tumor. **(f)** The representative Ki67 immunohistochemical staining image of the resected tumor. Scar bar, 100 μm.

Then 4 years later, the patient returned to our hospital for further treatment due to left upper limb paralysis, accompanied by severe neuropathic pain. The examination after admission showed that the VAS score of the patient was eight. The patient's muscle strength of left wrist flexion, forearm pronation, finger extension, and intrinsic hand muscles was grade 0, and that of left finger flexion, wrist extension, forearm supination, elbow flexion, elbow extension, shoulder abduction, and adduction was grade 3 (Figures 2a–d). Chest X-ray and CT showed multiple quasi-circular negative lesions in both lungs, which were considered metastatic lesions (Figures 2e, f), while the patient did not complain of dry cough or hemoptysis. Bone scanning suggested metastatic lesions in the left humeral head and left eighth rib (Figure 2g). In combination with the results of various examinations, the patient was diagnosed with recurrent MS after surgery. Considering that the severe neuropathic pain of the patient might be related to a nerve tumor, the tumor could be resected surgically to reduce his pain. Due to distant metastasis, this surgery aimed at tumor reduction but not complete resection. During the surgery, it was found that from the clavicle to the elbow, the median nerve was completely melanotic, and the ulnar, radial, and musculocutaneous nerves were partially melanotic (Figures 3a, c). Exploration of the roots and trunks of the brachial plexus revealed that the upper, middle, and lower trunks of the brachial plexus were completely melanotic, but the suprascapular nerve was not melanotic (Figure 3b). When the melanotic epineurium was cut open, the melanotic nerve fiber bundles could be observed. Among them, there were also not completely melanotic nerve fiber bundles. A nerve fiber with melanotic two ends, but a normal middle part, was also found. All these phenomena suggest the growth pattern of the tumor spreading along the nerve fibers (Figure 3d). Intraoperatively, most of the completely melanotic nerves that were visible were palliatively resected, and the cut ends of these nerves were protected. After surgery, the patient's pain was significantly relieved, and the VAS score was reduced to three. The postoperative pathological analysis showed that the melanin-enriched tumor cells were extensively proliferated and accompanied by focal necrosis. The Ki67 immunohistochemical staining was positive, which was consistent with the previous pathological results (Figures 3e, f). It was recommended that the patient should be transferred to the Department of Oncology for further treatment, but he returned to a local hospital for subsequent treatment due to personal reasons. At the 3-month and half-a-year follow-up, the patient's neuropathic pain could be controlled to a tolerable level by taking pregabalin 150–225 mg two times daily. Finally, the patient passed away 1 year after surgery.

## Discussion

Due to the small number of cases, there is no recognized treatment guideline or clinical experience for MS. Consequently, in the reported cases, the long-term therapeutic effect may not be ideal, but it still accumulates valuable experience for better treatment of this tumor in the future. It has been reported that PMS is one of the manifestations of Carney syndrome and has a malignant tendency (3). In the recently reported cases, recurrence and metastasis have also been found in patients with MS of non-psammomatous type and without Carney syndrome. To better analyze our case, we summarized the treatment and prognosis of MS cases similar to this case, which occurred in the peripheral nerve trunk and did not present Carney syndrome, so as to provide a basis for analyzing the treatment of this disease (Table 1). It was found that although only a few of these cases had tumor recurrence, most of the cases were followed up for a relatively short time, which may lead to many recurrences not being observed. Therefore, because of its uncertain biological features and potential recurrence risk, the treatment of MS should be carefully selected.

**Table 1:** Summary of the treatment of melanotic schwannoma cases without Carney complex.

**References**	**Location**	**Gender**	**Age**	**Nationality**	**Symptom**	**Duration**	**Treatment**	**Follow-up**	**Recurrence**
Hall et al. (4)	S1	Female	18	USA	Radicular pain	Several years	Resection + radiotherapy	2.5 years	No
Yeom et al. (5)	T11	Male	72	South Korea	Lower back pain, paresthesia	6 months	Resection	1 year	No
Georgiev et al. (6)	L3	Male	61	Bulgaria	Lower back pain	3 months	Resection	14 months	No
Soyland et al. (7)	T8	Male	53	USA	Acute chest pain	2 days	Resection	6 months	No
Morgan et al. (8)	C8	Female	50	UK	Tinel's sign	Several years	Resection	3 months	No
Solomou et al. (9)	C6	Female	45	UK	Neck pain, paresthesia	1 year	Resection + immunotherapy	15 months	Yes (death)
Shen et al. (10)	L3	Female	29	China	Lower back pain	1 day	Chemotherapy	1 year	No
Nagashima et al. (11)	S2	Male	48	Japan	Left sciatic pain	6 months	Resection	6 months	No
Kim et al. (12)	Facial nerve	Female	14	USA	Right facial weakness	No mention	Resection + chemotherapy	9 years	Yes
Azarpira et al. (13)	L2	Male	37	Iran	Lower back pain	8 months	Resection	4 months	No
Carrasco et al. (14)	Trigeminal nerve	Female	34	Chile	Odontalgia, hypoesthesia	2 months	Resection	3 months	Yes
Sengoz et al. (15)	S1	Male	41	Turkey	Radicular pain	14 months	Resection	6 months	No
Kuchelmeister et al. (16)	C6	Female	53	Germany	Brachialgia	2 years	Resection	1 year	No
Cummings et al. (17)	S2	Male	51	USA	Lower back pain	8 months	Needle biopsies	No mention	No mention

Although most literature reports that 90% of MS are benign, and only about 10% are recurred and metastasized, this does not mean that we can take such cases lightly. It is precisely this uncertainty that brings difficulties in the selection of treatment for such tumors (18). Among them, MS occurring in the peripheral nerve is different from those in other locations in terms of treatment. If the MS of the peripheral nerve trunk is benign, the treatment principle should be similar to that of common schwannomas. Common schwannomas usually do not lead to the paralysis of neurological function. Thus, the treatment principle for schwannomas with (or without) neurostimulation symptoms is to protect the nerve fiber bundles during tumor resection, and not damage nerve fibers, causing iatrogenic neurological impairment after surgery. In the resection of common schwannomas, the tumor tissue can usually be completely dissected from the nerve bundles, so as not to damage nerve fibers. However, benign MS occurring in the peripheral nerve trunk that can be dissected has not been reported.

In our case, because of the invasion of MS cells into the nerve fiber bundles, the nerve fiber bundles would be damaged during tumor resection, resulting in the loss of neurological function, which is a problem that must be fully considered when making a treatment plan for MS. If it is a benign MS, the first principle of treatment is to resect the tumor as thoroughly as possible without damaging the neurological function. When the tumor invades the nerve fibers and it has to remove partial nerve bundles, it is necessary to ensure the primary nerve repair or reconstruction as much as possible. The treatment of malignant MS should be completely different. The first principle of its treatment should be to resect the tumor completely and extensively and to dissect the surrounding lymph nodes and adjacent tissues, so as to prolong the survival of patients as much as possible. As for the loss of neurological function, it should not be repaired in the primary phase, but in the secondary phase after the tumor was completely resected, there was no recurrence. Based on the above principles, another problem puzzling the treatment of MS is how to determine whether it is benign or malignant. The first difficulty lies in the diagnosis of MS before cutting open the skin and exposing the tumor during surgery. Although some reports have suggested the imaging features of MS, these features are still not specific enough to enable us to distinguish MS from common schwannomas before surgery. Moreover, due to the low incidence, it is unreasonable to conduct a needle biopsy before the resection of all schwannomas for excluding the possibility of MS before surgery. Therefore, the diagnosis of MS is often an emergency during surgery. The second difficulty is that even if MS is found during surgery, we cannot determine whether it is benign or malignant. It is difficult to accurately determine the benign and malignant nature of tumor cells only by histological staining and cell morphology during the frozen pathological examination. The accurate diagnosis still depends on the immunohistochemical analysis. A series of molecular markers of the malignant tumor could be used to decide whether the tumor was malignant, such as Ki67, C-erbB, PCNA, NSE, Vim, and NESTIN. In this case, immunohistochemical analysis showed that the tumor cells were positive with Vim, H3K27m, Pan-mel, S-100, and Ki67, with indicated malignancy.

Based on this, we have reasons to make the following therapeutic decisions, that is, when we detect MS during surgery, the tumor should be completely resected, and the adjacent tissues and lymph nodes should be dissected according to the tumor invasion. Further treatment should be decided according to the pathological condition after surgery. If it is benign, nerve repair should be performed as soon as possible. In case of malignancy, radiotherapy or molecular targeted therapy should be continued, and nerve repair or functional reconstruction should be carried out after the tumor is completely eradicated.

In addition to prolongating the survival period, relieving the patient's severe neuropathic pain is also one of the important purposes of treatment. During the operation of this patient, we found that the invasion of melanin schwannoma resulted in swelling and stiffening of brachial plexus trunks and increased intra-nerve pressure, which may be one of the causes of neuropathic pain. Therefore, the epineural release of the diseased nerves, reducing the pressure within the nerves, can partially relieve the nerve pain.

## Conclusion

This article reports a rare case of malignant MS in the peripheral nerve trunk. Combined with the retrospective analysis of similar cases, a new idea on the treatment of MS in the peripheral nerve trunk is discussed and proposed, which will help to better understand the treatment of this rare nerve tumor in the future.

## Data availability statement

The datasets presented in this article are not readily available because of ethical and privacy restrictions. Requests to access the datasets should be directed to the corresponding authors.

## Ethics statement

The studies involving human participants were reviewed and approved by Xijing Hospital. The patients/participants provided their written informed consent to participate in this study. Written informed consent was obtained from the individual(s) for the publication of any potentially identifiable images or data included in this article.

## Author contributions

HW wrote the manuscript. LS and TT summarized the literature. ZS took the photographs. RC and RZ revised the manuscript. All authors contributed to the article and approved the submitted version.
